# Association of Early Norepinephrine Administration With 24-Hour Mortality Among Patients With Blunt Trauma and Hemorrhagic Shock

**DOI:** 10.1001/jamanetworkopen.2022.34258

**Published:** 2022-10-07

**Authors:** Tobias Gauss, Justin E. Richards, Costanza Tortù, François-Xavier Ageron, Sophie Hamada, Julie Josse, François Husson, Anatole Harrois, Thomas M. Scalea, Valentin Vivant, Eric Meaudre, Jonathan J. Morrison, Samue Galvagno, Pierre Bouzat

**Affiliations:** 1 Anesthesia and Critical Care, Grenoble Alpes University Hospital, Grenoble, France; 2 Program in Trauma, R Adams Cowley Shock Trauma Center, University of Maryland School of Medicine, Baltimore; 3 Sant’Anna School of Advanced Studies, Pisa, Italy; 4 Emergency Department, Lausanne University Hospital and University of Lausanne, Lausanne, Switzerland; 5 Department of Anesthesia and Critical Care, Hôpital Européen Georges Pompidou, AP-HP, Université de Paris, Paris, France; 6 Centre de Recherche en épidémiologie et Santé des populations, INSERM U 10-18, Université Paris-Saclay, Paris, France; 7 National Institute for Research in Digital Science and Technology (INRIA), Montpellier, France; 8 Institut Agro, Université Rennes, French National Centre for Scientific Research, Institut de recherche mathématique de Rennes, Rennes, France; 9 Department of Anesthesiology and Critical Care, Bicêtre Hospital, AP-HP, University Paris Saclay, Le Kremlin Bicêtre, France; 10 Cap Gemini Invent, Issy-Les-Moulineaux, France; 11 Department of Intensive Care Unit and Anesthesia, Military Teaching Hospital Sainte-Anne, Toulon, France; 12 University Grenoble Alpes, INSERM, U1216, CHU Grenoble Alpes, Grenoble Institute Neurosciences, Grenoble, France

## Abstract

**Importance:**

Hemorrhagic shock is a common cause of preventable death after injury. Vasopressor administration for patients with blunt trauma and hemorrhagic shock is often discouraged.

**Objective:**

To evaluate the association of early norepinephrine administration with 24-hour mortality among patients with blunt trauma and hemorrhagic shock.

**Design, Setting, and Participants:**

This retrospective, multicenter, observational cohort study used data from 3 registries in the US and France on all consecutive patients with blunt trauma from January 1, 2013, to December 31, 2018. Patients were alive on admission with hemorrhagic shock, defined by prehospital or admission systolic blood pressure less than 100 mm Hg and evidence of hemorrhage (ie, prehospital or resuscitation room transfusion of packed red blood cells, receipt of emergency treatment for hemorrhage control, transfusion of >10 units of packed red blood cells in the first 24 hours, or death from hemorrhage). Blunt trauma was defined as any exposure to nonpenetrating kinetic energy, collision, or deceleration. Statistical analysis was performed from January 15, 2021, to February 22, 2022.

**Exposure:**

Continuous administration of norepinephrine in the prehospital environment or resuscitation room prior to hemorrhage control, according to European guidelines.

**Main Outcomes and Measures:**

The primary outcome was 24-hour mortality, and the secondary outcome was in-hospital mortality. The average treatment effect (ATE) of early norepinephrine administration on 24-hour mortality was estimated according to the Rubin causal model. Inverse propensity score weighting and the doubly robust approach with 5 distinct analytical strategies were used to determine the ATE.

**Results:**

A total of 52 568 patients were screened for inclusion, and 2164 patients (1508 men [70%]; mean [SD] age, 46 [19] years; median Injury Severity Score, 29 [IQR, 17-36]) presented with acute hemorrhage and were included. A total of 1497 patients (69.1%) required emergency hemorrhage control, 128 (5.9%) received a prehospital transfusion of packed red blood cells, and 543 (25.0%) received a massive transfusion. Norepinephrine was administered to 1498 patients (69.2%). The 24-hour mortality rate was 17.8% (385 of 2164), and the in-hospital mortality rate was 35.6% (770 of 2164). None of the 5 analytical strategies suggested any statistically significant association between norepinephrine administration and 24-hour mortality, with ATEs ranging from –4.6 (95% CI, –11.9 to 2.7) to 2.1 (95% CI, –2.1 to 6.3), or between norepinephrine administration and in-hospital mortality, with ATEs ranging from –1.3 (95% CI, –9.5 to 6.9) to 5.3 (95% CI, –2.1 to 12.8).

**Conclusions and Relevance:**

The findings of this study suggest that early norepinephrine infusion was not associated with 24-hour or in-hospital mortality among patients with blunt trauma and hemorrhagic shock. Randomized clinical trials that study the effect of early norepinephrine administration among patients with trauma and hypotension are warranted to further assess whether norepinephrine is safe for patients with hemorrhagic shock.

## Key Points

**Question:**

Is norepinephrine administration associated with 24-hour mortality among patients with blunt trauma and hemorrhagic shock?

**Findings:**

In this cohor study of 2164 patients with blunt trauma and hemorrhagic shock from the US and France, according to 5 distinct statistical simulations, the average treatment effect showed no association between norepinephrine administration and 24-hour mortality.

**Meaning:**

This study suggests that norepinephrine administration is not associated with 24-hour mortality among patients with blunt trauma and hemorrhagic shock.

## Teaser

This cohort study uses data from 3 registries in the US and France to evaluate the association of early norepinephrine administration with 24-hour mortality and in-hospital mortality among patients with blunt trauma and hemorrhagic shock.

## Introduction

Uncontrolled hemorrhage remains the leading cause of preventable death among patients with trauma^1,2^ despite considerable improvements in trauma care.^3,4,5^ Trauma-induced hemorrhage and hypovolemia can trigger an intricate neurohormonal response.^6^ This hypotension is not explained by hypovolemia alone but is associated with sympathoinhibitory-induced vasodilation.^7^ It appears intuitive that vasopressor administration for patients with trauma and hypotension could play a role in treating vasoplegia and sustaining adequate perfusion pressure.

Multiple retrospective studies report that vasopressor infusion for patients with trauma is associated with increased mortality,^8,9,10^ which has led to conflicting practices in which early administration of vasopressors is either uncommon or discouraged in many North American trauma systems,^11,12,13^ whereas it is recommended in European guidelines on the management of bleeding.^14^ Considering these differences, we investigated the association of early norepinephrine administration, prehospital and in the resuscitation room, with 24-hour mortality among a cohort of patients with trauma, hypotension, and hemorrhagic shock. We hypothesized that norepinephrine administration would not be associated with a significant difference in 24-hour mortality.

## Methods

### Setting and Cohort

This retrospective, multicenter cohort study collected data from 3 regional trauma system registries; the details of each have been previously described: TraumaBase (Clichy, France),^15^ Trauma System of the Northern French Alps Emergency Network (TRENAU, Grenoble, France),^16^ and the R Adams Cowley Shock Trauma Center (RACSTC, Baltimore, Maryland)^17^ according to the Utstein template.^18^ The TRENAU and TraumaBase obtained institutional review board (Comité de Protection des Personnes, University of Paris VI, Paris, and Université Grenoble Alpes, France) approval from the Advisory Committee for Information Processing in Health Research (Comite Consultatif Pour le Traitement de l’information en matière de recherche dans le domaine de la santé CCTIRS, 11.305bis and 15.038bis) and from the National Data Protection Agency (Commission Nationale de l’Informatique et des Libertés CNIL, 911461 and CNIL 915372). For the RACSTC, the University of Maryland institutional review board approved the study. Informed consent was waived because the data were deidentified, and the patients or next of kin were informed and given the opportunity to oppose data use. This study followed the Strengthening the Reporting of Observational Studies in Epidemiology (STROBE) reporting guideline. This study was registered with ClinicalTrials.gov (NCT04497155).

### Inclusion Criteria

All consecutive patients with trauma admitted between January 1, 2013, and December 31, 2018, to each of the 3 participating registries were screened for inclusion. Inclusion was a 2-step process and was assessed retrospectively from registry data. The first step targeted all patients with hypotension; the inclusion criteria were as follows: being 18 to 89 years of age, having sustained an exclusively blunt mechanism of injury, and presenting with a prehospital or trauma center admission systolic blood pressure less than 100 mm Hg and/or a trauma center admission systolic blood pressure less than 100 mm Hg with or without norepinephrine. Blunt trauma was defined as any exposure to nonpenetrating kinetic energy, collision, or deceleration. Patients younger than 18 years, pregnant patients, and those who sustained a prehospital cardiac arrest were excluded. The second step targeted all patients in a hemorrhagic situation who required at least 1 of the following surrogate criteria: prehospital or resuscitation room administration of packed red blood cells, need for procedural hemorrhage control (including interventional radiology), massive transfusion of blood products (defined as >10 units of packed red blood cells) in the first 24 hours after admission, or death from hemorrhage. Fifteen international trauma experts agreed on these criteria in a Delphi process (eAppendix 4 in Supplement 1). None of these experts participated in the study group.

### Intervention and Outcome

Prehospital care was provided based on guidelines that have been previously published for the TraumaBase and TRENAU cohorts^19,20^ and are publicly available for the RACSTC cohort.^21^ The intervention group (norepinephrine) was documented as an exposure event with the administration of a continuous infusion of norepinephrine in the prehospital setting or in the trauma resuscitation bay. The decision to administer norepinephrine was made by the attending physician in the prehospital environment or the resuscitation bay in the TraumaBase and TRENAU cohorts according to European guidelines.^14^ Norepinephrine administered continuously via electrical syringe pumps without any bolus and no other vasopressor or inotrope was used throughout the study as the standard way to administer norepinephrine in France.^22^ Norepinephrine, prehospital blood products, and tranexamic acid were not available or administered to the RACSTC cohort. Intrahospital management and hemorrhage control were at the discretion of the attending trauma physician. The primary outcome was 24-hour mortality, starting with hospital admission. The secondary outcome was in-hospital mortality.

### Identification of Confounding Variables

Confounding variables expected to be associated with both the likelihood of exposure to the intervention group (norepinephrine) and the outcomes of interest were identified by a Delphi process, consisting of a group of 15 international experts in the field of trauma care and mapped into a directed acyclic graph^23^ (eAppendix 4 in Supplement 1).

### Management of Missing Data and Missing Attributes

Missing data were handled by either imputing the missing values according to 2 imputation algorithms or by explicitly accounting for missing values in the ATE estimation without imputation. The strategies to impute missing values were either based on factorial analysis for mixed data and consisted of performing a single imputation with a regularized iterative factorial analysis for mixed data model or performing a multivariate imputation by chained equations imputing missing entries. eAppendix 3 in Supplement 1 provides detailed information on the handling of missing values.

### Sensitivity Analysis

Considering missing data or differences in health care systems and populations in the US and French cohorts, the investigators performed several sensitivity analyses to rule out specific effects. Sensitivity analyses or robustness checks were performed first to assess the effect of missing data according to the 3 different approaches, second to assess only patients with hypotension, and third to assess only French patients. eAppendix 2 in Supplement 1 details these robustness checks.

### Statistical Analysis

Statistical analysis was performed between January 15, 2021, and February 22, 2022. Data are presented as absolute count and percentage for categorical variables, and continuous data are described as median and IQR values or mean and (SD) values. Categorical data were compared using the χ^2^ test or the Fisher exact test. Continuous data with a parametric distribution were evaluated with the Mann-Whitney test or the Wilcoxon rank sum test. The analysis was performed on the entire study population stratified by exposure group (ie, norepinephrine vs control). All *P* values were from 2-sided tests and results were deemed statistically significant at *P* < .05. The statistical package R, version 4.0 (R Group for Statistical Computing) was used for the entire analysis.

The mean association of norepinephrine administration with 24-hour mortality was estimated as the average treatment effect (ATE), according to the Rubin causal model (eAppendix 1 in Supplement 1). In this observational cohort study, confounding by indication was a threat to validity owing to the inherent differences in the patients and treatments that were compared. Inverse propensity weighting (IPW) was used to ensure that patients treated or not treated with norepinephrine were evenly distributed. Although multiple regression and propensity score–based methods have been found to lead to similar study conclusions, propensity score matching and IPW aim to achieve a balanced distribution of known confounders across treatment groups and to better emulate the properties of a randomized clinical trial.^24^ The lack of norepinephrine use in the RACSTC cohort represented an empirical violation of the positivity assumption, a key component of the Rubin causal model.^25,26^ The adopted approach compensated for this violation (eAppendix 1 in Supplement 1). The ATE was estimated with a corresponding 95% CI by IPW and the doubly robust approach.^27,28^ Inverse propensity weighting measures the confounding variables associated with the treatment assignment and outcome and assigns a sample weight as the inverse of the propensity of the observed sample. In contrast, the doubly robust method integrates additional variables associated with patient outcome. Norepinephrine administration was considered to be associated with either a significant increase or decrease in 24-hour mortality if both extremes of the 95% CI were above or below zero, respectively. If the 95% CI estimate crossed zero, the data would indicate that norepinephrine was not significantly associated with 24-hour mortality. The variance of the ATE was computed through appropriate estimators or bootstrapping.

Several simulation scenarios were used to account for the empirical violation of the positivity assumption and applied to the data set (eAppendix 1 in Supplement 1). Simulation trials identified 5 strategies to reduce the ATE bias due to the empirical violation of the positivity assumption and compared these with a strategy without any correction. Strategy 1 was an ATE estimation without any correction. Strategy 2 was an ATE estimation in the combined TraumaBase, TRENAU, and RACSTC cohorts through regression adjustment, with 2 distinct models: one for the norepinephrine group and one for the control group. Strategy 3 was an ATE estimation in the combined TraumaBase, TRENAU, and RACSTC cohorts through weighted regression adjustment, with 2 distinct models: one for the norepinephrine group and one for the control group and weighting all patients in the RACSTC cohort according to their similarity with patients in the control group in the TraumaBase and TRENAU cohorts. Strategy 4 was an ATE estimation in the RACSTC cohort matching each patient in terms of baseline confounders with a patient in the norepinephrine group from the TraumaBase and TRENAU cohorts, corresponding to standard propensity score matching. Strategy 5 was an ATE estimation in the RACSTC cohort matching each patient with a patient in the norepinephrine group from the TraumaBase and TRENAU cohorts according to a univariate measure of similarity, represented by the estimated probability of belonging to the RACSTC cohort given baseline covariates.

Strategies 4 and 5 allowed for an ATE estimation in the RACSTC cohort. The global estimation of ATE is achieved by combining these results with the estimated ATE from the TraumaBase and TRENAU cohorts.

## Results

**Figure 1.**
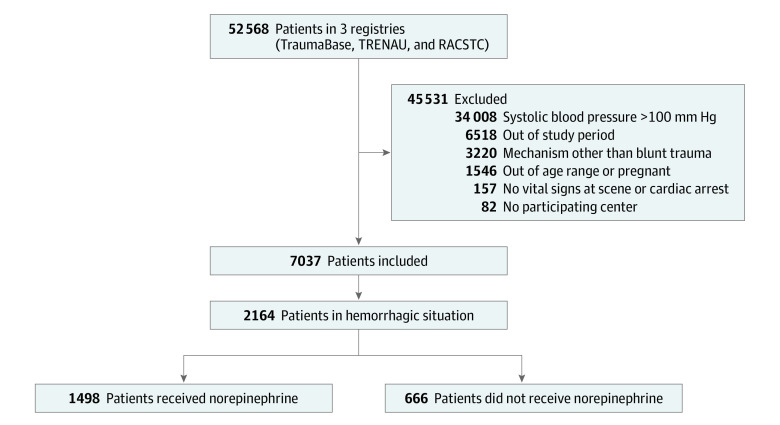
Flow Diagram of Study Participation RACSTC indicates R Adams Cowley Shock Trauma Center; and TRENAU, Trauma System of the Northern French Alps Emergency Network.

A total of 52 568 patients were screened for inclusion in the study (25 081 in the TraumaBase cohort, 7151 in the TRENAU cohort, and 20 336 in the RACSTC cohort), and 2164 (1508 of men [70%]; mean [SD] age, 46 [19] years; median Injury Severity Score, 29 [IQR, 17-36]; 1449 patients [67%] had ≥1 body region with an Abbreviated Injury Scale score of ≥3) presented with acute hemorrhage and were included for analysis (Table). Figure 1 provides a Consolidated Standards of Reporting Trials (CONSORT) diagram of patients included in the final study analysis. A total of 1497 patients (69%) required emergency hemorrhage control measures, 128 (6%) received a prehospital transfusion of packed red blood cells, and 543 (25%) received a massive transfusion. Norepinephrine was administered to 1498 patients (69%). The administration of norepinephrine was distributed as follows: 1212 of 1429 patients (85%) in the TraumaBase cohort, 286 of 380 patients (75%) in the TRENAU cohort, and none in the RACSTC cohort. Overall 24-hour mortality was 18% (385 of 2164); 24-hour mortality was comparable between the 3 cohorts (RACSTC, 56 of 355 [16%]; TRENAU, 64 of 380 [17%]; TraumaBase, 265 of 1429 [19%]). In-hospital mortality was 36% (770 of 2164).

**Table.** Characteristics of Patients

Characteristic	Patients, No. (%)			
	TraumaBase (n = 1429)	TRENAU (n = 380)	RACSTC (n = 355)	Total (N = 2164)
Age, mean (SD), y	45.0 (19.6)	46.0 (18.9)	49.0 (19.7)	46.0 (19.0)
Sex				
Female	464 (32.4)	72 (18.9)	100 (28.2)	656 (30.3)
Male	965 (67.5)	288 (75.8)	255 (71.8)	1508 (69.6)
Prehospital total transport time, mean (SD), min	99.0 (55.3)	102.0 (43.3)	76.0 (118.9)	92.0 (72.0)
Ground transportation	1101 (77.0)	200 (52.6)	147 (41.4)	1448 (66.9)
Prehospital				
Systolic blood pressure, mean (SD), mm Hg	100.0 (35.0)	100.0 (31.9)	99.0 (31.5)	100.0 (31.0)
Heart rate, mean (SD), beats/min	100.0 (35.6)	95.0 (32.0)	101.0 (30.7)	98.0 (32.0)
Intubation	938 (65.6)	176 (46.3)	0	1114 (51.0)
Motor GCS score, median (IQR)	6.0 (1.0-6.0)	6.0 (3.0-6.0)	5.0 (1.0-6.0)	6.0 (1.0-6.0)
Blood products	93 (6.5)	35 (9.2)	0	128 (5.9)
Norepinephrine	809 (56.6)	162 (42.6)	0	971 (44.8)
Admission				
Systolic blood pressure, mean (SD), mm Hg	95.0 (33.0)	86.0 (33.9)	96.0 (36.8)	92 (34.0)
Heart rate, mean (SD), beats/min	100.0 (33.4)	92.0 (34.3)	98.0 (37.2)	96 (34.0)
GCS score when not sedated, median (IQR)	14.0 (6.0-15.0)	13.0 (3.0-15.0)	13.0 (3.0-15.0)	13.3 (3.0-15.0)
Transfusion in resuscitation room	1098 (76.8)	228 (60.0)	314 (88.5)	1640 (75.7)
Norepinephrine started in resuscitation room	350 (24.5)	175 (46.0)	0	525 (24.2)
Transfusion of all blood products per 24 h, mean (SD), L	8.3 (7.7)	4.7 (6.3)	10.3 (11.4)	8.2 (8.5)
Massive transfusion^a^	357 (24.9)	83 (21.8)	103 (29.0)	543 (25.0)
ISS, median (IQR)	33.0 (22.0-43.0)	29.0 (20.0-41.0)	27.0 (17.0-36.0)	29.0 (17.0-36.0)
AIS head score, median (IQR)	2.0 (0.0-4.0)	2.0 (0.0-5.0)	2.0 (0.0-3.0)	2.0 (0.0-3.0)
24-h Mortality	265 (18.5)	64 (16.8)	56 (15.8)	385 (17.8)
ICU length of stay, mean (SD), d	15.0 (21.2)	21.0 (20.7)	10.0 (16.1)	15 (19.0)
In-hospital mortality	547 (38.3)	115 (30.2)	108 (30.4)	770 (35.6)

Abbreviations: AIS, Abbreviated Injury Scale; GCS, Glasgow Coma Scale; ICU, intensive care unit; ISS, Injury Severity Score; RACSTC, R Adams Cowley Shock Trauma Center; TRENAU, Trauma System of the Northern French Alps Emergency Network.

^a^ More than 10 units of packed red blood cells per 24 hours.

### Association of Norepinephrine With 24-Hour Mortality

**Figure 2.**
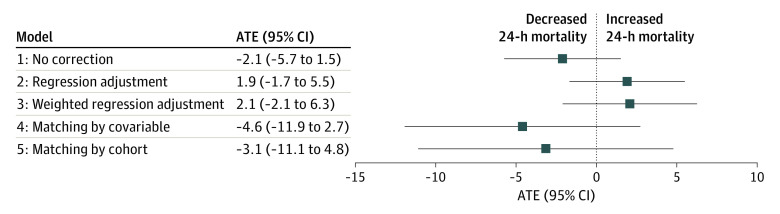
Average Treatment Effect (ATE) Estimation of Association of Norepinephrine With 24-Hour Mortality Model 1: ATE estimation in the combined US and French cohort with no correction. Model 2: ATE estimation in the combined US and French cohort through regression adjustment. Model 3: weighted regression adjustment for all untreated patients and US patients weighted according to their similarity with untreated French patients. Model 4: ATE estimation in the US cohort matching each US patient with a treated French patient with similar baseline confounders combined with ATE estimate in the French cohort to generate a global ATE in the combined cohorts (US and French). Model 5: ATE in the US cohort matching each US patient with a treated French patient with a similar probability of belonging to the US cohort given confounders combined with ATE estimate in the French cohort to generate a global ATE in the combined cohorts (US and French). Models used the doubly robust approach and multivariate imputation by chained equations.

The ATE estimation in the combined US and French cohorts (strategy 1) showed neither a significant association with 24-hour mortality with no correction (ATE, −2.1; 95% CI, −5.7 to 1.5) nor a significant association with 24-hour mortality after regression adjustment (strategy 2; ATE, 1.9; 95% CI, −1.7 to 5.5) or weighted regression adjustment (strategy 3; ATE, 2.1; 95% CI, −2.1 to 6.3) (Figure 2). Estimation in the US cohort matching each US patient with a treated French patient with similar baseline confounders (strategy 4) generated no association with 24-hour mortality (ATE, −4.6; 95% CI, −11.9 to 2.7). This result is similar to the ATE according to strategy 5, matching each US patient with a treated French patient with a similar probability of belonging to the US cohort according to confounders (ATE, −3.1; 95% CI, −11.1 to 4.8). In summary, none of the 5 strategies suggested any statistically significant association between norepinephrine administration and 24-hour mortality.

### Association of Norepinephrine With In-Hospital Mortality

**Figure 3.**
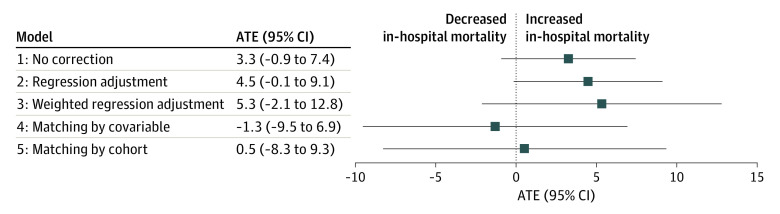
Average Treatment Effect (ATE) Estimation of Association of Norepinephrine With In-Hospital Mortality Model 1: ATE estimation in the combined US and French cohort with no correction. Model 2: ATE estimation in the combined US and French cohort through regression adjustment. Model 3: weighted regression adjustment for all untreated patients and US patients weighted according to their similarity with untreated French patients. Model 4: ATE estimation in the US cohort matching each US patient with a treated French patient with similar baseline confounders combined with ATE estimate in the French cohort to generate a global ATE in the combined cohorts (US and French). Strategy 5: ATE estimate in the US cohort matching each US patient with a treated French patient with a similar probability of belonging to the US cohort given confounders combined with ATE estimate in the French cohort to generate a global ATE in the combined US and French cohorts. Models used the doubly robust approach and multivariate imputation by chained equations.

After adjustment for treatment assignment and outcome and imputation of missing data, no association with in-hospital mortality could be shown with any of the 5 strategies (strategy 1: ATE, 3.3; 95% CI, –0.9 to 7.4; strategy 2: ATE, 4.5 95% CI, –0.1 to 9.1; strategy 3: ATE, 5.3; 95% CI, –2.1 to 12.8; strategy 4: ATE, –1.3; 95% CI, –9.5 to 6.9; strategy 5: ATE, 0.5; 95% CI, –8.3 to 9.3) (Figure 3). All 95% CIs in all approaches crossed the numerical zero value, suggesting no association of norepinephrine administration with hospital mortality.

### Sensitivity Analysis

Three sensitivity analyses (ie, robustness checks) assessed the association of missing data with outcomes, the association of missing data with outcomes among all patients with hypotension, and the association of missing data with outcomes among the French cohort. In all 3 analyses, there was no association between norepinephrine and 24-hour mortality (eAppendix 2 in Supplement 1).

## Discussion

In this retrospective cohort study, early norepinephrine administration was not significantly associated with 24-hour or in-hospital mortality among patients with blunt trauma, hypotension, and hemorrhagic shock. To our knowledge, the present study is the largest investigation on the use of vasopressors after traumatic, hemorrhagic shock and further contributes to the literature on early norepinephrine administration for patients with trauma and hypotension.

The findings in this study contrast with previous studies.^28^ A multicenter study from the collaborative on inflammation and the host response to injury concluded that vasopressors within 12 hours of injury were associated with increased mortality.^13^ The study examined a composite exposure to any vasopressor, such as phenylephrine, dopamine, vasopressin, and norepinephrine. A single-center study reported similar findings for 1349 patients, independent of volume status.^12^

In both studies,^2,13^ various pharmacologic agents with very different cardiovascular effects were pooled as vasopressors. Furthermore, the criteria and timing to initiate these agents were not specified. Patients who died within 48 hours were excluded, thereby eliminating those most likely to die from exsanguination. In contrast, in a propensity score–matched study, norepinephrine administration within 24 hours of injury for patients receiving early packed red blood cells was not associated with increased 24-hour or in-hospital mortality.^9^

Emerging data have supported the biological plausibility of using norepinephrine for patients with trauma and hemorrhagic shock.^9^ Hemorrhage is a time-dependent process, necessitating early recognition and management. Observations from humans demonstrate a biphasic response that begins with a sympathoexcitatory phase of vasoconstriction frequently followed by a subsequent sympathoinhibitory phase,^29^ which suggests that clinical deterioration in the various forms of shock reflect a unifying mechanism associated with vascular dysfunction and hyporesponsiveness.^7,30^ Norepinephrine is a β-1 and α-1 agonist that is superior to phenylephrine (pure α-1 agonist) in maintaining regional blood flow in patient populations at risk for bleeding and hypoperfusion. Recent data from an animal model showed improved survival after norepinephrine infusion, irrespective of a lower or higher blood pressure goal.^31^

Previous observational studies and reviews concluded that increased mortality was associated with vasopressor use among patients with trauma, but these studies and reviews investigated a heterogenous mix of agents without concise clinical policy.^32,33^ In the present cohort, only norepinephrine was administered under physician supervision, according to longstanding routine use supported by clinical guidelines in France.^22,34^ Norepinephrine was not administered under any prehospital jurisdiction or during acute hemorrhage management in the resuscitation bay in our RACSTC cohort, providing a coherent control group. The higher rate of prehospital intubation in the French cohort might require more norepinephrine administration to compensate for sedation-induced vasodilation. Compared with vasopressin,^10^ norepinephrine is more widely available and less expensive.

### Strengths and Limitations

This study has some strengths. It collected data from multicenter, international cohorts in high-volume trauma systems. Statistical analyses included multiple adjustments for confounding variables with inverse probability weighting and doubly robust methods to assess patients who were exposed to early norepinephrine. The selection of confounding variables, as well as the definition of patients at risk of hemorrhage, was performed a priori by a Delphi process of international experts in trauma care. The primary outcome of 24-hour mortality is consistent with recent statements that encourage investigating clinically significant and relevant outcomes in the trauma population, other than in-hospital or 28-day mortality.^35^ Furthermore, we also included patients who were at risk of death owing to hemorrhage and excluded those considered to be unsalvageable (eg, patients with blunt trauma and prehospital cardiac arrest with potential administration of epinephrine). Ultimately, our findings should be interpreted strictly within the context of the available data; early norepinephrine administration was not associated with a significant increase or a significant decrease in 24-hour mortality among patients with blunt trauma, hypotension, and hemorrhage.

There are numerous limitations to this study. The most important limitation consists of the violation of the positivity assumption because patients in the RACSTC cohort did not receive norepinephrine. To account for this limitation, we used several strategies that resulted in similar end points, suggesting the robustness of our approach. A random error measurement and regression dilution bias cannot be excluded with regard to the blood pressure measurement being associated with the inclusion criteria and confounders.^36^ The possibility of unidentified and residual confounding exists, despite attempts undertaken in an international Delphi process with detailed confounder mapping (directed acyclic graph). By including exclusively patients who were admitted alive, a risk of survivor bias remains. Hamada et al^37^ provide an estimation for the TraumaBase cohort of 60% of deaths occurring on the scene after a car accidents. Our results may not necessarily apply to all injury patterns, in particular penetrating injuries, traumatic brain injury, or spinal cord injury. Maintaining a target perfusion pressure may be particularly beneficial for these injury types, and this issue should be explored. Nonetheless, the characteristics of the cohort are representative of many US and European populations of patients with blunt trauma, and the approach assumes that the association between the outcome and the covariates are the same in all the cohorts.

Our study was not designed to evaluate organ function or subsequent multiple organ failure. Prospective randomized clinical trials demonstrated that early vasopressin infusion for trauma patients with hemorrhage is efficacious and results in lower volumes of blood product transfusion without an increase in mortality or complications, providing further evidence to contradict that vasopressors should be avoided in patients with trauma.^10,38^ Vasopressin infusions were not available in the prehospital environment in any of our study cohorts, nor were vasopressin infusions administered in the resuscitation bay prior to emergency hemorrhage control.

We were unable to investigate the association of a specific blood pressure threshold or the volume of administered crystalloid fluid expansion with mortality. Although previous animal and human studies suggest that early vasopressor use is associated with decreased resuscitation volume,^10,39^ it was a considerable limitation in our investigation because crystalloid volumes were associated with multiorgan failure among severely injured patients, and vasopressors should be administered only after fluid expansion. Because of the retrospective nature of our study, we were unable to decipher the precise reason for norepinephrine use for each patient. Although administration of norepinephrine was under the direct supervision of a physician in both the prehospital setting and the resuscitation bay in the French cohorts, it is unclear why certain patients received norepinephrine and others did not.

## Conclusions

In this multicenter cohort study, early use of norepinephrine was not associated with increased mortality among patients with blunt trauma and hemorrhagic shock, challenging the dogma that vasopressors should be avoided after traumatic injury. Considering both the complex pathophysiology of hemorrhage and the conflicting evidence in the literature, there is a need for multicenter studies to investigate and clarify the potential clinical association of early vasopressor administration with mortality among patients with trauma and hemorrhagic shock.

## Notes

Supplement 1. **eAppendix 1.** Causal Inference Approach **eAppendix 2.** Robustness Checks **eAppendix 3.** Explorative Analysis **eAppendix 4.** Delphi to Identify Confounding Factors

Supplement 2. **Nonauthor Collaborators.** French Trauma Research Initiative
