# Supplementary material for: Association of Early Norepinephrine Administration With 24-Hour Mortality Among Patients With Blunt Trauma and Hemorrhagic Shock
Source: JAMA Netw Open. 2022 Oct 7;5(10):e2234258. doi: 10.1001/jamanetworkopen.2022.34258 (PMC9547317; doi:10.1001/jamanetworkopen.2022.34258)
Supplement: Supplement 1. — eAppendix 1. Causal Inference Approach eAppendix 2. Robustness Checks eAppendix 3. Explorative Analysis eAppendix 4. Delphi to Identify Confounding Factors [file jamanetwopen-e2234258-s001.pdf]

Supplementary Online Content

Gauss T, Richards JE, Tortù C, et al; French Trauma Research Initiative. Association of early norepinephrine administration with 24-hour mortality among patients with blunt trauma and hemorrhagic shock. *JAMA Netw Open.* 2022;5(10):e2234258. doi:10.1001/jamanetworkopen.2022.34258

eAppendix 1. Causal Inference Approach
eAppendix 2. Robustness Checks
eAppendix 3. Explorative Analysis
eAppendix 4. Delphi to Identify Confounding Factors

This supplementary material has been provided by the authors to give readers additional information about their work.

© 2022 Gauss T et al. *JAMA Network Open.*

eAppendix 1. Causal Inference Approach

e1.1 Principles of causal inference

Causal inference studies intend to estimate the causal effect of an intervention on an outcome. Such studies are highly diffused in all fields of social sciences, such as Economics, Political Sciences and Health. In health sciences, a causal inference study may be designed to assess the effect of a new drug (intervention) on mortality (outcome). In the present study, causal inference methodologies are employed to give an answer to the following research question: does the exposition to Noradrenaline (NOR) reduce the risk of death in shocked trauma patients in hemorrhage?
More technically, the goal of any causal inference assessment is to estimate the so-called Average Treatment Effect (ATE), which measures the average impact of the intervention on the outcome over the population of interest. To pursue this goal, the most popular methodological framework for causal inference studies - the Rubin Causal Model - follows a theoretical approach based on potential outcomes, which represent the potential values of the outcome under each level of the intervention. The individual treatment effect results by the comparison of the individual potential outcomes. The main issue is that the individual potential outcomes cannot be simultaneously observed, as each agent can be either assigned to the active intervention (i.e the new drug) or to the control intervention (i.e the placebo). Therefore, any causal inference evaluation faces a missing data issue, where the researcher has to find the best strategy to impute the missing potential outcome.
Formally, a causal inference evaluation takes into account the following quantities:

- N = population of interest, made up by N individuals (each individual is indexed as i)
- W_i= treatment assignment. By convention W_i=1 corresponds to the active treatment, while W_i=0 to control or no treatment.
- Y_i(1) , Y_i(0) real-valued couple: potential outcomes, Y_i(0) corresponds to the response of i when no treatment is given, Y_i(1) to the response of I when treatment is given.
- Y_i real-valued: observed outcome of unit i. The observed outcome is the potential outcome corresponding to the actual treatment assignment.
- X_i vector: covariates or feature vector that contain additional information about an agent. Specifically, for each individual i, this vector includes the information on K characteristics, which are not affected by the intervention. These characteristics are likely to affect i) the treatment assignment, ii) both the treatment assignment and the outcome.

e1.2 Observational Studies

In the empirical scenario considered in this work, the intervention of interest (the NOREPI administration) is not randomly assigned to patients. The treatment assignment mechanism is not known, since the doctors deliberately choose whether to administer NOREPI to their patients (formally, the study on the effect on NOREPI on mortality is an *observational study*). This issue slightly complicate the statistical analysis. As it is well grounded in the literature on policy evaluation studies. the absence of a proper randomization design may introduce dependencies between treatment and potential outcomes and unbalancies between the treated group and the untreated group in terms of pre-treatment covariates. This issues, if not properly accounted for, may compromise the analysis and lead to an inaccurate estimate of the global ATE. For instance, If an observational study compares two groups of patients, the group receiving the treatment associates most often with a higher level of severity and thus carries a higher probability to receive the treatment. The control of confounding factors that at the same time attribute administration of the treatment and/or influence the outcome becomes crucial in order to balance these factors between the two groups in the absence of randomization.

© 2022 Gauss T et al. *JAMA Network Open.*

The identification of principal confounding factors culminates in the construction of a causal relationship model. These confounding factors are defined by an association to the outcome criterion but are not supposed to contribute to the causal pathway between the exposition variable and the outcome [1]. The causal model summarizes the various hypotheses about the eventual causal relationships to define the analysis plan based on the available scientific knowledge. In this study, confounders were identified by a Delphi process consulting with an international group of 15 experts in trauma (see section Appendix IV for list of experts, Delphi questions and results). Pre-intervention (prior to the administration of NOR) variables identified by the Delphi process concerned factors that would influence the clinician to administer NOR (e.g. haemorrhage, see figure 3.1). In the final model (see section doubly robust method) all variables associated to the severity of the shock and 24-hour mortality (e.g. Glasgow Coma Scale) as well as criteria associated with the treatment administration were mapped with the program dagitty (http://dagitty.net) into a Directed Acyclic Graph (DAG, section 4, figure 4.1).

From the methodological point of view, accounting for the baseline information included in the confounding factors is useful to frame the observational setting ressemble as much as possible to a randomized trial: if the baseline information is relevant, conditioning on that the observational study has similar properties of a randomized setting. Therefore, the information contained in the data set allows this compensation in order to emulate an experiment by reversing or retracing the treatment assignment process.

In order to estimate the ATE from an observational data set, a correction is required to compensate for the lack of randomization of the treatment assignment (in the present example NOREPI or no NOREPI). First of all, conditioning on prior information, the researcher should be able to credibly assume the absence of any dependency between the intervention and the potential outcomes: this assumption - known as *unconfoundedness* - is the key assumption of any causal evaluation in an observational setting, as it allows the researcher to estimate the Average Treatment Effect. This assumption can be translated as "there is sufficient information about the treatment assignment decision process captured in the covariates, so that conditionally on every value for, we have random treatment assignment, implying independence between potential outcomes and treatment assignment". The unconfoundedness assumption cannot be neither tested or validated: it should be discussed, given the empirical setting and the available pre-treatment covariates. The process of identifying those factors that are likely to affect the treatment or the outcome (or both) is called identifiability and, in the case of observational data, deciding whether these factors are sufficient for relying on the unconfoundedness requires domain-specific knowledge. The information required should allow to emulate an experiment by reversing or retracing the treatment assignment process.

Following the *unconfoundedness* assumption, all confounding factors, i.e., all factors that drive both the treatment assignment (e.g. shock) and the outcome (e.g. mortality) require to be observed before the treatment assignment. Some other factors are likely to affect either the treatment or the outcome: only those factors affecting the outcome only can be observed even after the treatment assignment. If the unconfoundedness assumption is likely to hold, conditioning on covariates, the Average Treatment Effect can be outlined and fairly estimated, by implementing an estimation strategy that accounts for these covariates.

© 2022 Gauss T et al. *JAMA Network Open.*

As previously outlined, pre-treatment characteristics are also useful to balance treated and untreated units. In the randomized scenario, the balance in terms of baseline information is guaranteed by the treatment assignment itself, while in the observational setting this is not true, as the randomization plan does not even exist. Therefore, it is essential to condition on pre-treatment characteristics in observational studies, so to make the treatment assignment becoming as good as random. This process of correcting for the nonrandomized treatment assignment is also called *deconfounding* and can be achieved by different means, namely by matching or weighting methods. The quality of this balancing can be assessed in different ways using for instance different statistical tests reference, but the most popular approach is to compare standardized mean differences before and after the balancing. If these differences fall beyond some small threshold, usually 10%, for all confounding factors, the balancing step is considered successful. If the balancing step is however failing or insufficient, then the balancing method or the propensity model need to be revisited.

Sometimes conditioning on the full set of covariates may be complicated, since, if the number of included characteristics is high, this could imply an high-dimensionality issue. To avoid this issue, researchers often prefer to employ an alternative measure - called *Propensity Score*-, which is an univariate synthesis of the individual covariates and represents the individual probability of being assigned to the active intervention, given covariates. This quantity can be fairly estimated by implementing a proper statistical model, chosen according to the characteristic of the intervention (for instance, the logit model can be employed if the intervention is binary). It has been proved by previous contributions in the literature on causal inference methods for observational studies that , if the study is unconfounded given the baseline covariates, then it remains unconfounded given the propensity score, which summarizes that covariates. Moreover, the propensity score is a balancing score which effectively reduces the imbalance between the two treatment groups.

e1.3 Empirical violation of the positivity assumption

In the present study, an additional methodological issue requires to be taken into account: the so called empirical violation of the positivity assumption. This issue emerges as in the US cohort none of the patients actually received NOREPI. Therefore, conditioning on the cohort information, in the specific sample we observe in the data, a US patient has a null probability of being assigned to the active intervention. This phenomenon implies that US doctors are more skeptical towards administering NOREPI. However we assume that in the entire population of US patients affected by an hemorrhagic shock there are some patients (units) who receive NOREPI (if this is true, the positivity assumption is *empirically violated* but not *theoretically violated*). The empirical violation of the positivity assumption may introduce a bias in the estimates. We have tested various methods to account for the violation in the positivity assumption in some simulated scenarios. The approaches compensating effectively for the estimation bias caused by the violation of the positivity assumption are based on *Regression Adjustment* and *Matching*.

Regression adjustment: We define two distinct regression models for treated and untreated individuals: in both models, we regress the outcome variable on confounders. Then, we predict for all individuals the potential outcome under treatment and control (using the estimated coefficients of the two models defined at the previous step). Finally, we compute the mean difference of the two imputed outcomes. We employ two different strategies for implementing the regression adjustment: i) a *standard regression adjustment*, where all units have the same weight; ii) a *weighted regression*

© 2022 Gauss T et al. *JAMA Network Open.*

adjustment, where we estimate the ATE on the observed sample after having assigned to the units in the US cohort an additional weight depending on their baseline similarity with the units in the French cohort. The last procedure implies some steps, that can be summarized as follows.

1) Obtain the mean baseline profile of an untreated unit in the French cohort: the idea is to look at the baseline characteristics of the untreated units in the French cohort and to derive the mean profile (practically, it means computing the mean of each covariate among the untreated in the French cohort).
2) Compute the similarity between each of the individuals in the US cohort with the mean baseline profile of an untreated unit in the French Cohort obtained at the previous step.
3) Estimate the ATE using weighted regression adjustment, where the weights are represented by the similarity measures obtained at step 2): note that all the units in the French cohort are weighted 1, while units in the US cohort have been assigned to weight which is between 0 and 1 (possibly included).

Matching-based strategy: We estimate the effect in the whole population using a matching-based approach: the procedure consists in two steps

1) we estimate the effect in the US cohort by considering a matched sample where each individual belonging to the US cohort is matched with the most similar treated patient referring to the Frech cohort. Similarity is computed according to either i) the baseline covariates or ii) the predicted probability of referring to the US cohort, given covariates. In the latter situation, we first define a regression model of the cohort variable on pre-treatment characteristics, and then we compute the predicted probability of referring to the US cohort given covariates (this measure is used as an univariate synthesis of covariates).

2) once obtained the estimated ATE for the US cohort we sum this measure with an estimate of the ATE for the first cohort (where no positivity issue has to be faced), so to obtain a global estimate of ATE for the whole population

As previously indicated, these methodologies have been tested in simulated scenarios: that are all designed to be as adherent as possible to the analysis of the present study. In particular, we simulate a study with the objective to estimate the effect of an intervention on a cohort of interest and the whole population is composed of by two sub-cohort: the first one (cohort 1) satisfies the positivity assumption both theoretically and empirically, in the second one (cohort 2) only theoretical positivity applies (empirical positivity is violated in the empirical data). Formally, our theoretical population *dat* includes two cohorts, named *dat1* and *dat2*, which both satisfy the positivity assumption. However, in the population we actually observe, the observed population *obsdat* only one of the two cohorts *obsdat1* satisfies the positivity assumption, while in the observed sample of the second cohort *obsdat2* the treatment status is set to 0 for all units. Both the theoretical and the observed sample provide information about some pre-treatment characteristics X.

Thus, in the simulation setting, the observed population has been obtained by taking into account the individuals belonging to the theoretical population, with their own characteristics, and by forcing all the units referring to the second cohort to be untreated. The idea is to simulate the treatment effect in the whole population, then observing how different approaches perform in estimating the average

© 2022 Gauss T et al. *JAMA Network Open.*

treatment effect in the observed sample, while reducing the estimation bias due to the violation of the positivity assumption. The effect is expected to be heterogeneous in both sub population with respect to observed covariates, and there is a distributional shift. The Data Generating Process (DGP), identifies reliably i) the target effect in the entire cohort (target), ii) the target effect in the first sample (target1), iii) the target effect in the second sample (target2) These values will be used as reference points in each of the techniques. We ran the simulated analysis 5000 times.

The resulting plot (eFigure 1.1) compares the distributions of the estimated ATE according to the different methodologies [in bracket we denote the measure that each distribution should target]. Specifically, it compares a) the estimated real in the theoretical population (DGP) [ref: target]. The estimated target ATE in the theoretical subsamples dat1 and dat2 separately [ref: target1 and target2]; c) the estimated ATE in the whole observed population, without any correction (no corr) [ref: target]; d),e) the estimated ATE in the whole observed population, obtained through regular regression adjustment (r reg) and weighted regression adjustment (w reg) [ref: target]; estimated ATE in the second cohort (s coh) and in the whole sample (w sam) obtained through matching with respect of covariates [ref: target2 and target, respectively]; estimated ATE in the second cohort and in the whole sample obtained through matching with respect of the probability to belong to the second cohort [ref: target2 and target, respectively]. The figure illustrates the proposed methodologies based on regression adjustment and matching all reduce the estimation bias due to the violation of the positivity assumption. The obtained results result is robust against the extent of the distributional shift between the covariates' distribution in the two sub-samples and the extent of the correlation among confounders (the results represented in Figure A Iare obtained under a strong distributional shift, with no correlation among confounders).

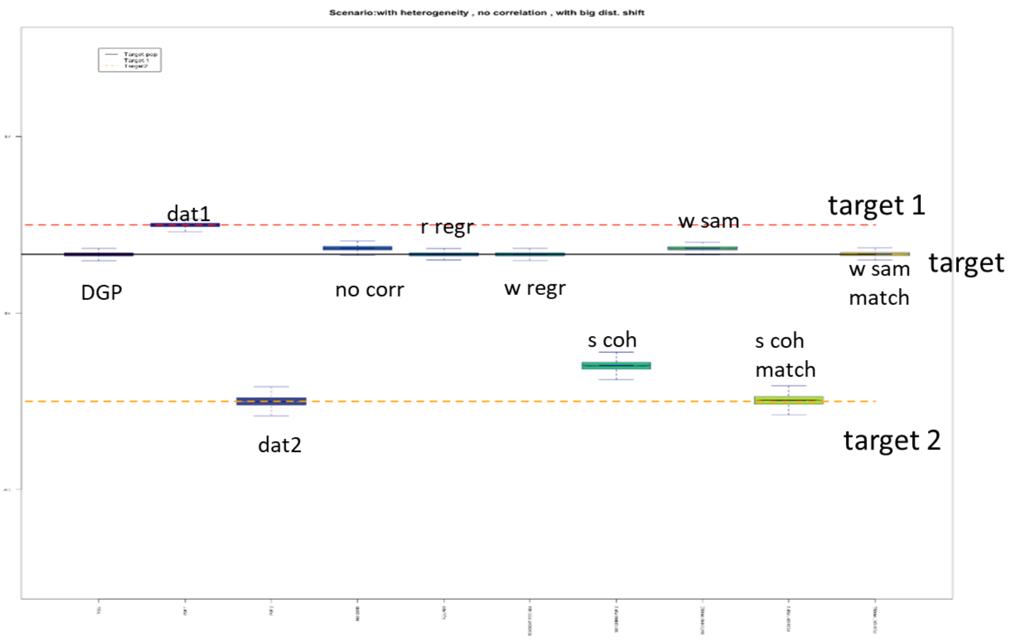

eFigure 1.1 , distributions of the estimated ATE according to the different methodologies; labels: DGP= theoretical population; dat 1/dat 2= theoretical samples; no corr= no correction; r regr= regular regression; w regr= weighted regression; s coh= second cohort; w sam= whole sample; s coh match=second cohort matched; w sample= whole sample matched

© 2022 Gauss T et al. *JAMA Network Open.*

Based on the results of the simulation analysis, we implemented the following strategies to compensate for the empirical violation of the positivity assumption:

- ATE estimation in the combined US and French cohort through regression adjustment, with two distinct models one for treated and one for untreated patients (Strategy 2)
- ATE estimation in the combined US and French cohort through weighted regression adjustment, with two distinct models one for treated and one for untreated and weighting all US patients according to their similarity with untreated French patients (Strategy 3)
- ATE estimation in the US cohort matching each US patient to a treated French patient with similar characteristics in terms of baseline confounders (Strategy 4)
- ATE estimation in the US cohort matching each US patient to a treated French patient according to a univariate measure of similarity, represented by the predicted probability to belong to the US cohort given baseline covariates (Strategy 5)

e1.4 ATE Estimation: Methodological approach

In the present study the treatment assignment mechanism is not known, since doctors decide whether to assign NOREPI to their patients. Therefore, the DAG identified by the Delphi process (see Supplementary Section 5), outlines which factors are likely to affect either the intervention or the outcome (or both) has to be used to estimate the Average Treatment Effect. The ATE estimation is performed by implementing several and heterogeneous methodologies, as to verify the robustness of results against different estimation strategies.

When no correction to compensate for the violated positivity was applied, the estimation approach differentiated with respect of a) the different strategy for dealing with missing data and b) the different strategies of estimation.

The missing values of the confounders can be either imputed or accounted for, without any imputation. The imputation of missing data can be performed by using two different approaches. The first approach consists in performing a single imputation with a (regularized) iterative Factorial Analysis for Mixed Data model. This model allows to account both for the similarities among individuals and for the statistical relationships among variables. The number of FAMD dimensions to be included is obtained by implementing a cross validation algorithm, over the incomplete data set (in this case, the number of included dimensions equals 3). In the second approach, we impute missing entries by using the Multivariate Imputation by Chained Equations (MICE). By applying this procedure, we generate several complete data sets, with no missing entries. The alternative solution for dealing with missing data, without explicitly imputing them is to estimate the propensity score using generalized random forests with Missing Incorporated Attributes.

The two estimators that employed to obtain an overall measure of ATE are the Inverse Probability Weighting (IPW) estimator and the Doubly-Robust (DR) Estimator. In the first scenario, ATE is computed by weighting units according to the value of the estimated propensity score. In the second scenario, the ATE is estimated by employing two different models, one for the treatment variable and one for the outcome variable: this last solution is more robust since it returns an unbiased estimate of ATE if at least one of the two models is well specified.

© 2022 Gauss T et al. *JAMA Network Open.*

When we explicitly account for the violation of the positivity assumption, we use all the methodologies proposed in the previous subsection - so the Regression Adjustment based methodologies and the Matching based methodologies-. This allows us to verify the robustness of results against different approaches to deal with the empirical violation of the positivity assumption.

© 2022 Gauss T et al. *JAMA Network Open.*

eAppendix 2. Robustness Checks

e2.1 Robustness for missing values

The raw data presented many missing entries, including variables to defining inclusion/ exclusion criteria. In consequence, we performed the following alternative analyses to the one presented in the main manuscript to double check the reliability of the obtained results when dealing with missing attributes.

1) *Restrictive approach*: We considered that all those units with missing values in a given variable concerning exclusion/inclusion criteria have not satisfied that criterion, according to the given variable.

2) *Imputation approach*: We first imputed missing attributes. In particular, before applying the inclusion-exclusion criteria, we imputed missing values in the variables determining these criteria by using the MICE R package (imputing missing attributes via chained equations). This imputed the *completed* dataset (no more missings on the variables of interest for the filtering/inclusion process), we estimated the inclusion-exclusion criteria on this dataset, retaining for each cohort only observations eligible to be included in the final sample.

3) *Loss approach*: We considered that all those units with a missing value in a given variable concerning exclusion/inclusion criteria have satisfied that criterion, according to the given variable. In the global analysis presented in the main of the manuscript a unit with a missing feature that determined the given criterion, it was not considered to meet the criterion of the given variable.

In the main manuscript, we presented results according to the *restrictive approach*. Here, we follow the second approach, based on the imputation of missing entries. We first imputed missing attributes which affect the variables determining the inclusion/exclusion criteria, using the very raw data, and then we applied the filtering process. Note that we perform the present analysis on the **French cohorts only,** as the raw data concerning the Baltimore cohort do not provide all the required information. The imputation is performed using MICE. By following this approach, the final sample included 5838 observations, 4679 from Traumabase and 1159 from TRENAU.

eFigure 2.1 illustrates the absence of any impact of Norepinephrine on 24-hours mortality, independently on the employed methodology.

© 2022 Gauss T et al. *JAMA Network Open.*

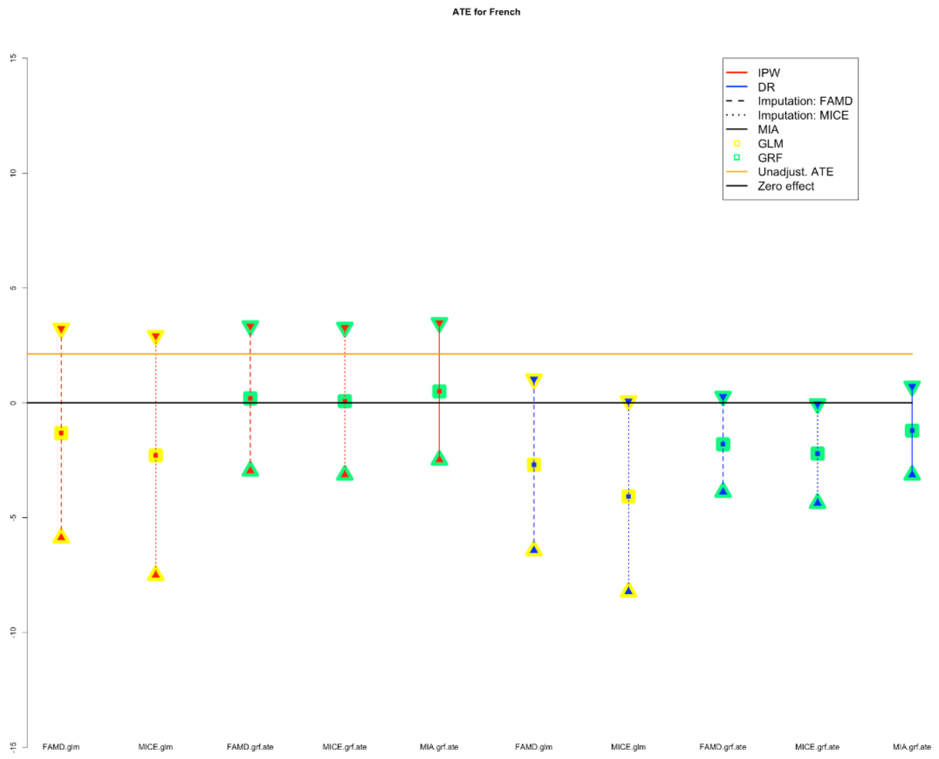

eFigure 2.1 Effect of NOREPI on 24-hour mortality according to the approaches employed

e2.2 Robustness for hypotensive patients

As a final robustness check, we performed an analysis including exclusively all hypotensive patients with a prehospital systolic arterial pressure < 100mmHg. The final sample consisted of 7837

© 2022 Gauss T et al. *JAMA Network Open.*

patients, 4679 from Traumabase, 1159 from TRENAU and 1199 from Baltimore. eFigure 2.2 demonstrates the lack of Norepinephrine on 24-hour mortality for all approaches employed.

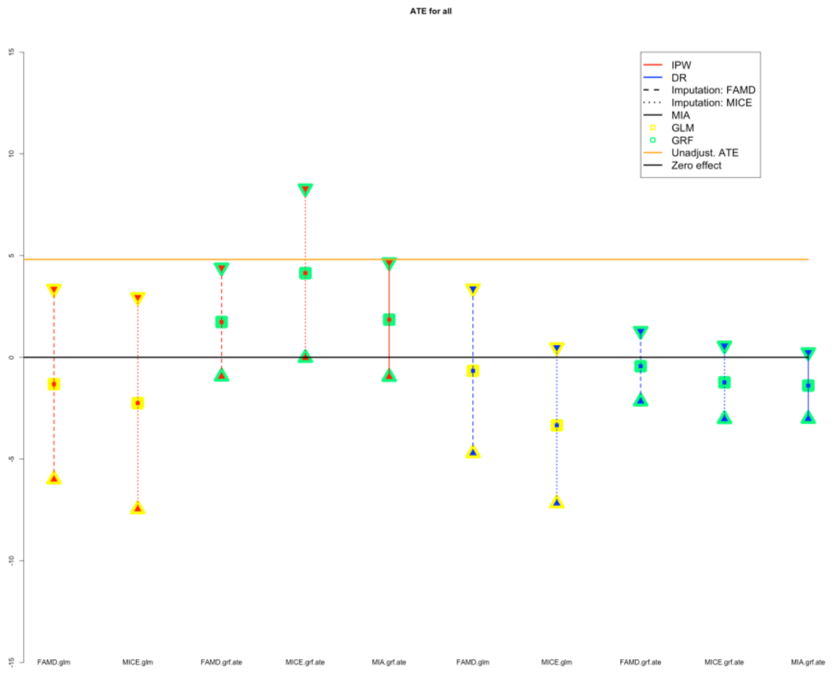

eFigure 2.2 Effect of Norepinephrine on 24-hour mortality for patients with prehospital systolic blood pressure < 100 mmHg

e2.3 Robustness for French cohort

Finally, we estimated the average treatment effect on the French cohort only, obtained under the first approach towards the missing entries in the variables determining the inclusion/exclusion criteria (the same adopted in the core of the paper).

The following eFigure 2.3 shows that norepinephrine has no significant impact on 24h mortality in the French cohort, independently on the employed statistical methodology.

© 2022 Gauss T et al. *JAMA Network Open.*

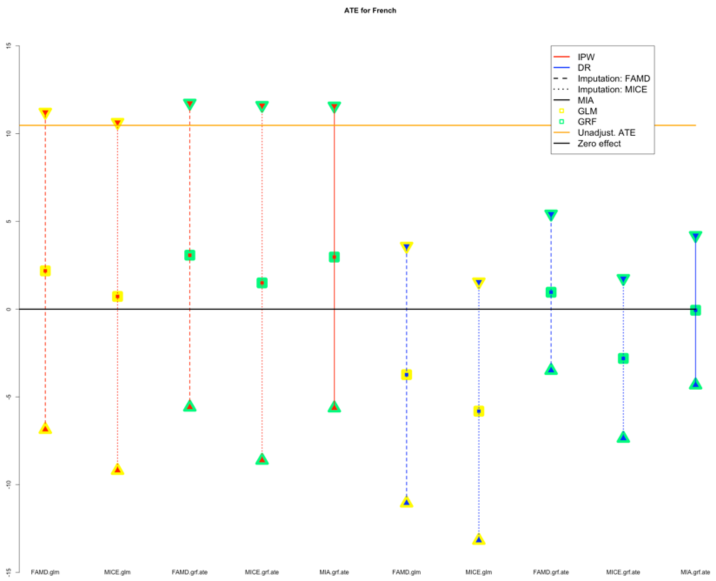

eFigure 2.3 Effect of Norepinephrine on 24-hour mortality in French cohort according to various methodological approaches

In summary: none of the robustness check performed for a) missingness, b) in hypotensive patients or c) exclusively in the French cohort showed a significant effect on 24-hour mortality.

© 2022 Gauss T et al. *JAMA Network Open.*

eAppendix 3. Explorative Analysis

e3.1 Covariates distributions among cohorts

In this section we show and discuss the distribution of the pre-treatment characteristics and their variation over the three included cohorts.

The following eFigure 3.1 is a matrixplot which provides information on the statistical distribution of the continuous confounders, in the whole sample: in particular, (i) the plots that lie on the diagonal of the matrix represent the univariate distribution of the continuous confounders, (ii) the graphs placed at the bottom-left triangle of the matrix show the scatterplots of each pair of confounders, (iii) while on the top-right triangle cells it is possible to observe the correlation and its significance.

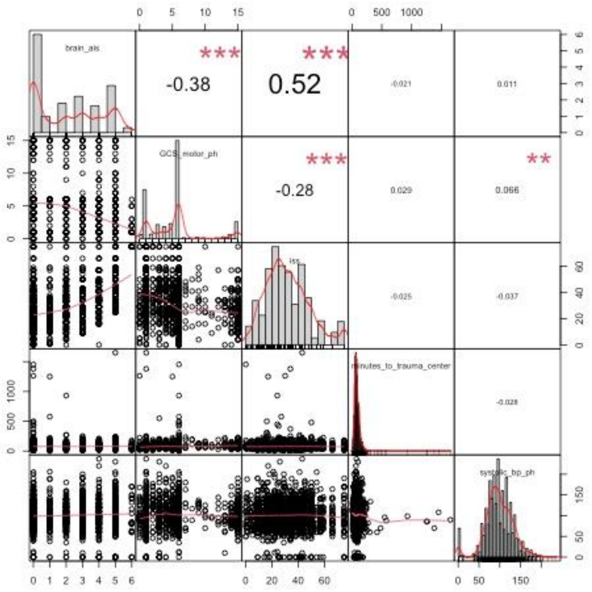

eFigure 3.1 Matrixplot. Univariate and joint distribution of continuous confounders, in the whole sample

The eFigure 3.2 provides the same information but considers the variation over the three different cohorts. Graphical elements are colored according to the cohort they refer to: blue curves refer to the TraumaBase cohort, red elements describe the TRENAU cohort, and green elements refer to the US cohort. Here even binary confounders are included (sex, transport type, and pre-hospital intubation), their distribution is represented through a bar plot.

© 2022 Gauss T et al. *JAMA Network Open.*

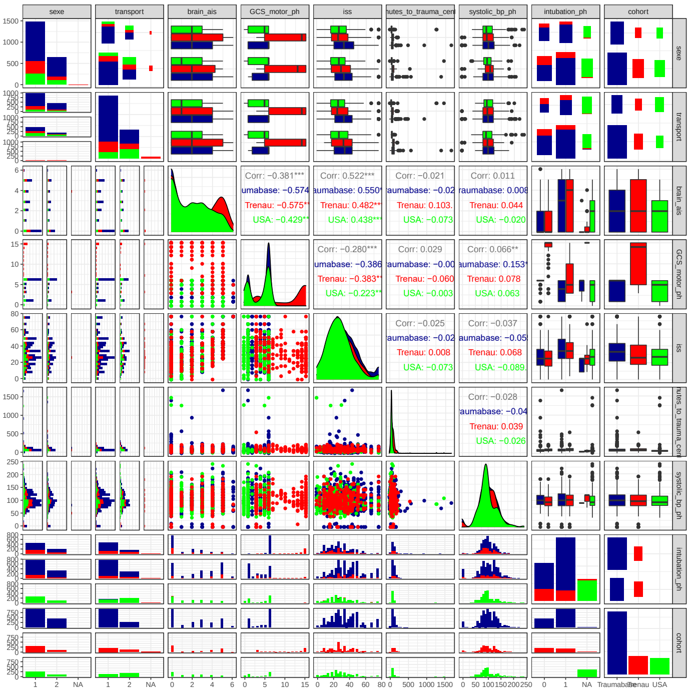

eFigure 3.2 Matrixplot. Univariate and joint distribution of continuous confounders, in the three cohorts

To explore the variation of the distribution of the confounders over the three cohorts, eFigure 3.3 explores whether the value of a given variable in a given cohort is significantly above the mean (red rectangles), below the mean (blue rectangles) or around the mean (white rectangles). Surprisingly, the TRENAU cohort appears to be more similar to the US cohort with respect of pre-treatment characteristics, than to the TraumaBase cohort.

© 2022 Gauss T et al. *JAMA Network Open.*

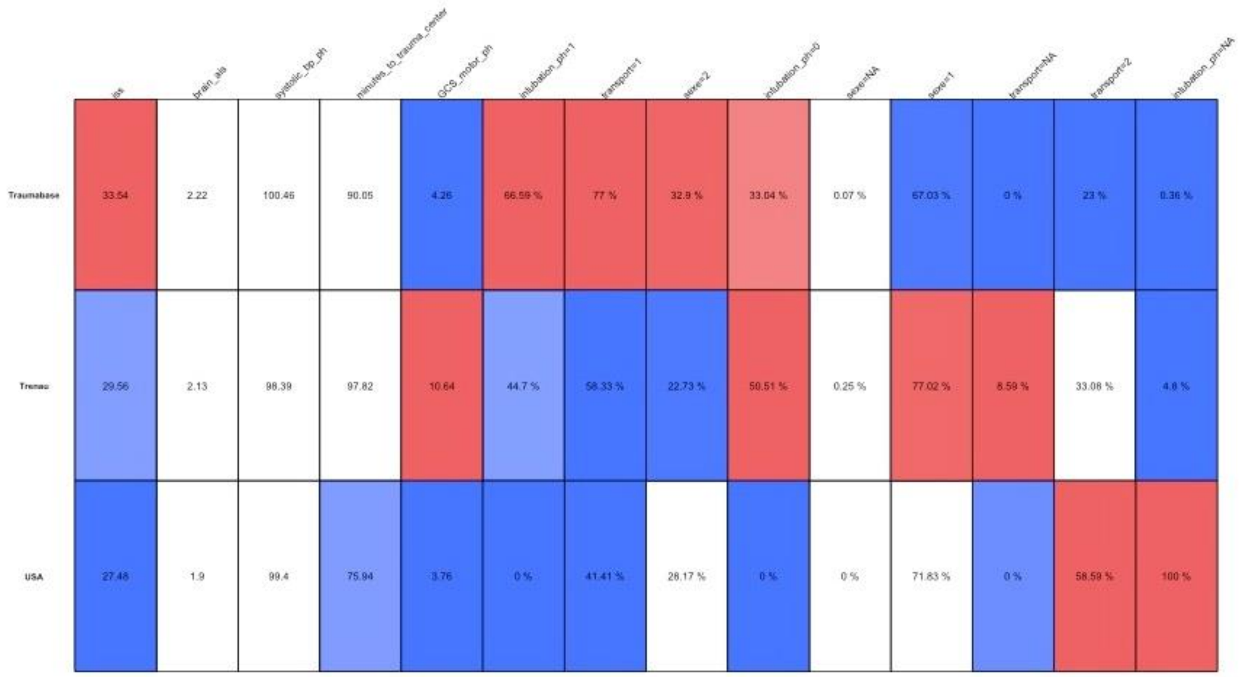

eFigure 3.3 Comparison of the distribution of the confounders, in the three cohorts. Red triangles signal value that significantly differ from the mean, white rectangles signal values that are close to the mean, blue rectangles signal value below the mean

Information on baseline characteristics and their variation over cohorts can be inspected in eTable 3.1, providing the mean and the standard deviation of each variable, in each cohort and the results of a statistical Two Sample t-test, which tests the hypothesis of no difference in means between the covariate distributions. The T-test comparison is implemented for the three pairwise comparisons of the cohorts. If the corresponding p-value of a given comparison is below 0.05, then the null hypothesis of no difference in means must be rejected and we can state that there is a significant difference between the covariate distribution in that examined comparison. The significant comparisons are signaled through stars (*) (one star refers to weekly significant comparisons*).

eTable 3.1 Distribution of pre-treatment characteristics, in the three examined cohorts. The table reports the mean and standard deviation of each variable in each cohort and the p-value of the two-sided T-test.

	Traumabase	Trenau	Usa	Traumabase vs Trenau	Traumabase vs USA	Trenau vs USA
	mean (sd)	mean (sd)	mean (sd)	p.value of the t.test	p.value of the t.test	p.value of the t.test
sexe	0.33(0.47)	0.23(0.42)	0.28(0.45)	0,00004 ***	0.07969*	0.09195*
transport	0.23(0.42)	0.36(0.48)	0.59(0.49)	2,551241 ***	3,056604E-25 ***	0,001254209 ***
brain_ais	2.22(2.04)	2.13(2.16)	1.9(1.73)	0.48857	0.00286 ***	0.09762
GCS_motor_ph	4.26(2.17)	10.64(5.02)	3.76(2.44)	3,310333E-70 ***	0.00059 ***	5,077743E-76 ***
iss	33.54(17.03)	29.56(14.77)	27.48(15.02)	0,00001 ***	0,0000108728 ***	0.05779*
minutes_to_trauma_center	90.05(83.03)	97.82(49.25)	75.94(119.05)	0.04258	0.04049 **	0.00196 **
systolic_bp_ph	100.46(35.2)	98.39(32.28)	99.4(31.54)	0.32359	0.59803	0.67852
intubation_ph	1.67(0.47)	1.47(0.5)	NaN(NA)	0,00000001164 ***	*NA*	*NA*

© 2022 Gauss T et al. *JAMA Network Open.*

e3.2 Covariates distribution in treated and untreated patients

In this section, we discuss the covariates distributions and their variation between treated and untreated patients.

The eFigure 3.4 shows the distributions of the eight included confounders, in the two treatment groups.

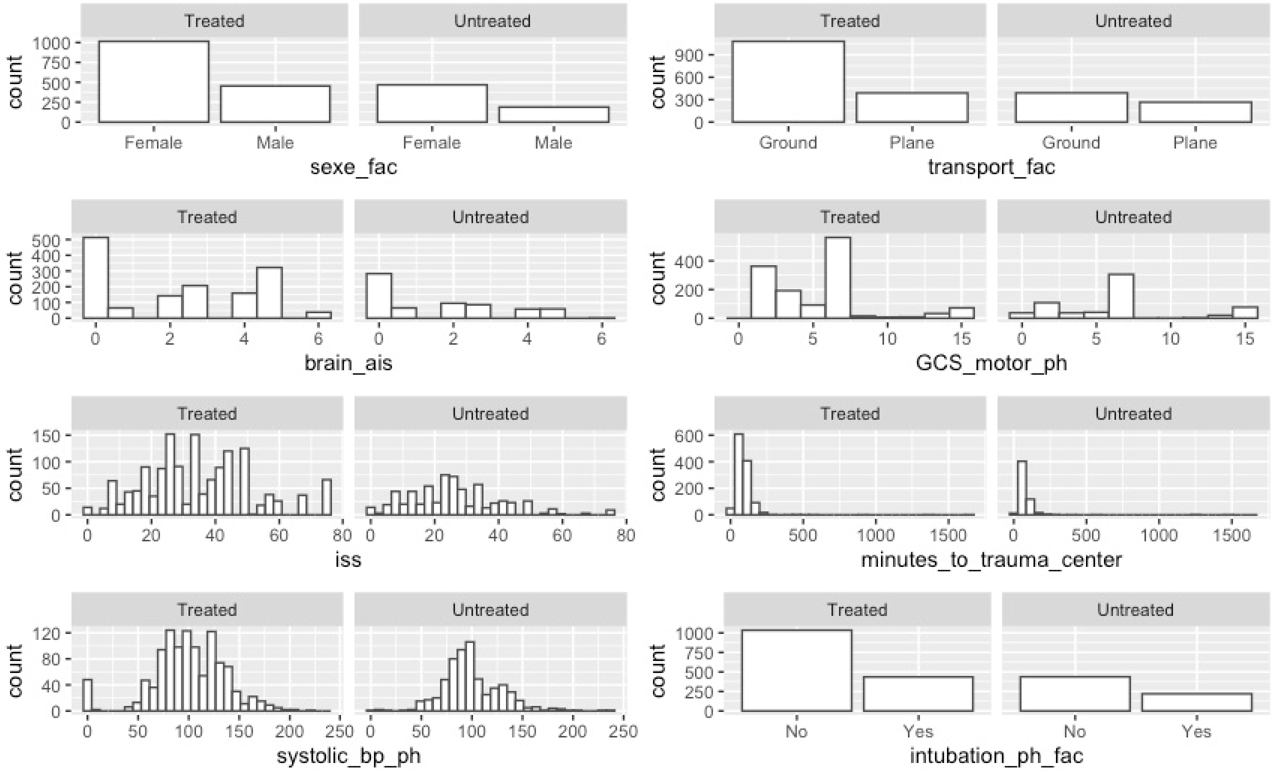

eFigure 3.4 Distribution of pre-treatment characteristics: treated vs untreated patients

Information on baseline characteristics and their variation over treated and untreated patients can be also examined by observing the following eTable 3.2, providing the mean and the standard deviation of each variable, in each treatment group and the results of a statistical Two Sample t-test, which tests the hypothesis of no difference in means between the covariate distributions. If the corresponding p-value is below 0.05, then the null hypothesis of no difference in means must be rejected and we can state that there is a significant difference between the covariate distribution between treated and untreated patients. The significant comparisons are signaled through stars (*) (one star refers to weekly significant comparisons*).

© 2022 Gauss T et al. *JAMA Network Open.*

eTable 3.2 Distribution of pre-treatment characteristics: treated vs untreated patients. The table reports the mean and standard deviation of each variable in each treatment group and the p-value of the two-sided T-test.

	Treated	Untreated	Treated vs Untreated
	mean (sd)	mean (sd)	p.value of the t.test
sexe	0.31(0.46)	0.29(0.45)	0.28497
transport	0.27(0.44)	0.41(0.49)	0,0002798122 ***
brain_ais	2.38(2.08)	1.62(1.75)	0,00000000001652813***
GCS_motor_ph	5.01(3.57)	5.97(4.33)	1.514974e-06***
iss	34.36(16.78)	25.97(14.18)	8,68666E-26 ***
minutes_to_trauma_center	91.55(80.05)	82.15(100.98)	0.05311 *
systolic_bp_ph	99.91(36.38)	99.71(28.88)	0.90171*
intubation_ph	0.31(0.46)	0.29(0.45)	0.28497

According to the Delphi agreement of the participating experts, age was exclusively analyzed as a factor to impact the outcome mortality but not treatment assignment. The results in the main manuscript reflect to this approach.

To demonstrate that inclusion of age as confounder for the outcome mortality and treatment assignment would have no impact on the results, we provide this secondary analysis including age as confounder for both outcome and treatment assignment (eFigure 3.5).

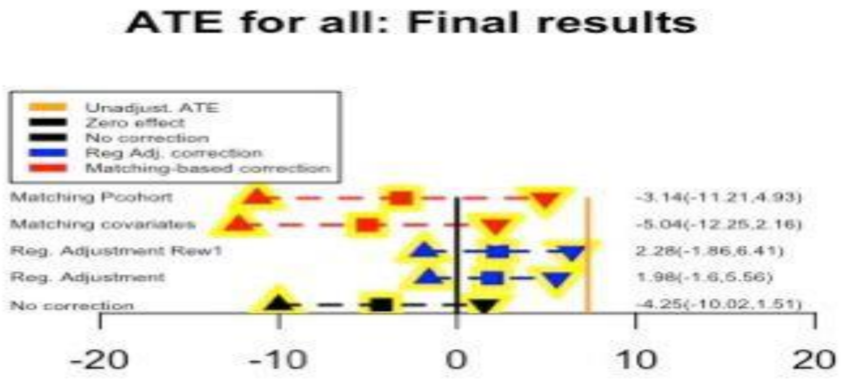

eFigure 3.5 Average Treatment effect for all patients on mortality with age included as confounder for outcome and treatment assignment

e3.3 Missing data analysis

Data present a relevant number of missing entries. This subsection provides a descriptive analysis of missing data, in the whole sample and in the single cohorts. This examination is focused on the confounding variables, related to the outcome and treatment allocation and on the post-treatment variables, which are likely to impact the outcome variable only.

© 2022 Gauss T et al. *JAMA Network Open.*

The eFigure 3.6a and b provide information on the distribution of missing cases: the left side graph show a representation of the distribution of the number of missing entries, while the right-side graph represents the cumulative sum of missing cases. As it is immediate to observe, the presence of missing entries represents a big issue for this study.

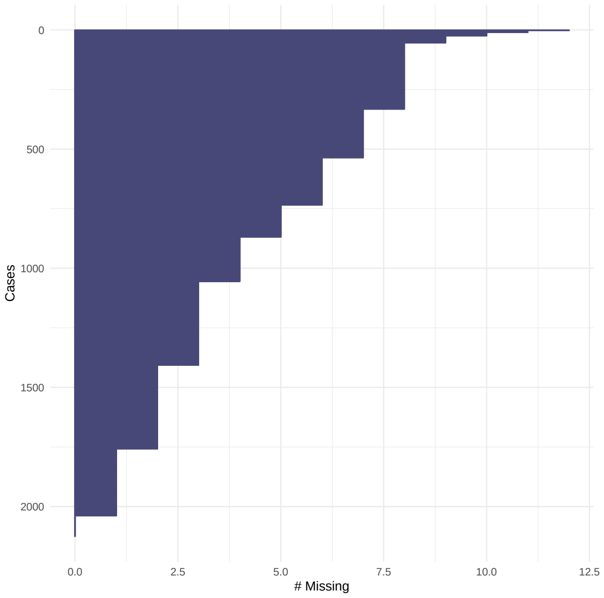

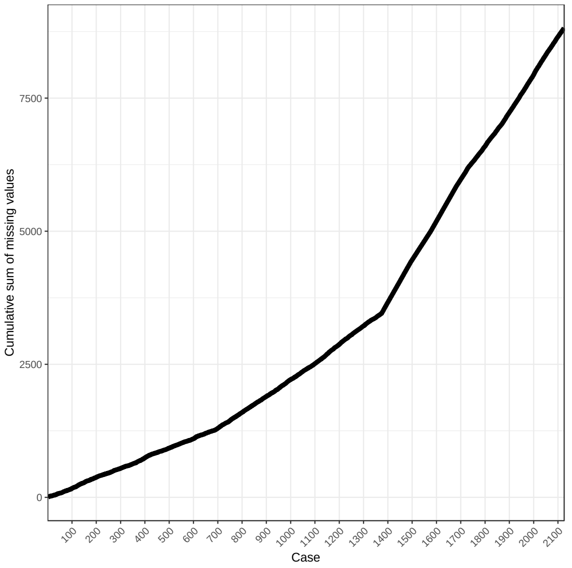

eFigure 3.6a and b Missing cases. Distribution of the number of missing entries (left), cumulative sum of missing cases (right)

The eFigures 3.7a and b show the percentage of missing values for each variable (left side figure) and the percentage of missing entries for each variable in the three cohorts (right side figure). The right-side graph suggests that some variables are not observed in some cohorts (yellow rectangles).

© 2022 Gauss T et al. *JAMA Network Open.*

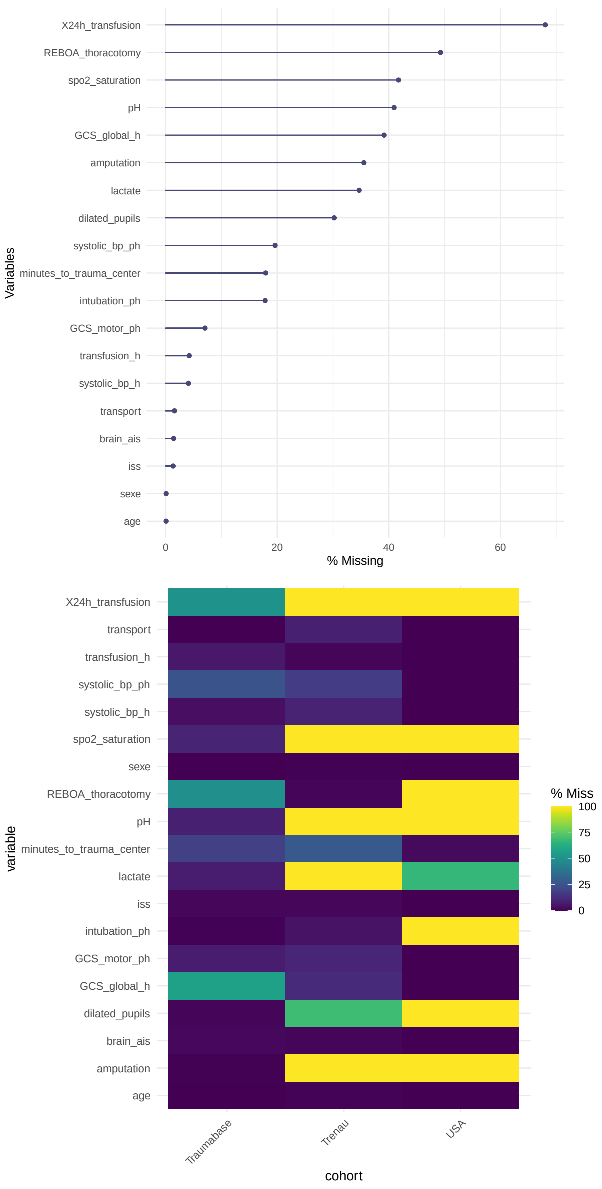

eFigure 3.7 a and b. Percentage of missing entries in the whole sample (left side figure) and in the three cohorts separately (right side figure*)*

The eFigure 3.8 a and b investigate common patterns of missingness. Moreover, they allow us to observe whether there is a significant association between the value of a variable and the missingness of another one. The left side graph is a matrix plot, where each rectangle represents a cell in the data matrix. Red rectangles signal missing entries, while observable entries are coloured according to a grey scale (the darker is the grey, the higher is the value of the given entry). Left-side Figure represents instead the number of missings in each variable and for certain combinations of variables (which tend to be missing simultaneously).

© 2022 Gauss T et al. *JAMA Network Open.*

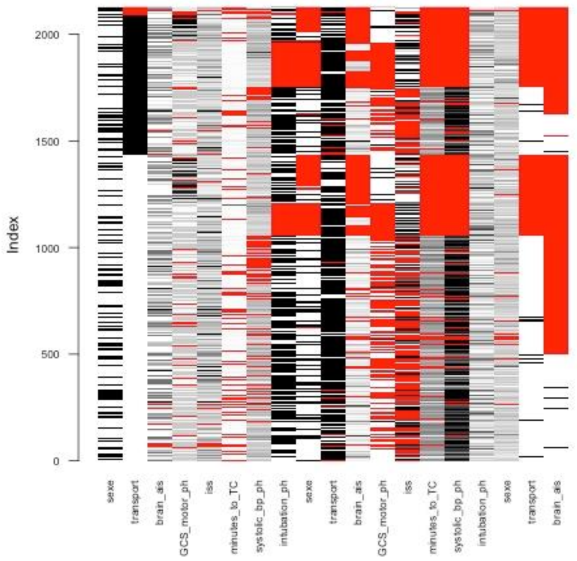

.

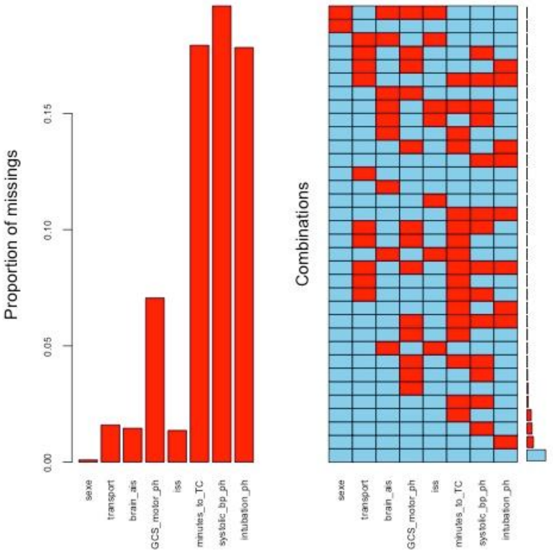

eFigure 3.8 a and b. Common patterns of missingness.

© 2022 Gauss T et al. *JAMA Network Open.*

eAppendix 4. Delphi to Identify Confounding Factors

Experts consulted:

Europe

De Backer, MD, PhD, Belgium

Martin Dünser, MD, PhD, Austria

Tim Harris, MD, PhD, United Kingdom

Anatole Harrois, MD, PhD, France

Marc Leone, MD, PhD, France

Eric Meaudre, MD, PhD, France

Julien Pottecher, MD, PhD, France

Jacob Steinmetz, MD, PhD, Denmark

Kjetil Sunde, MD, PhD, Norway

Karim Tazarourte, MD, PhD, France

USA

Richard P Dutton, MD, MBA

David V Feliciano, MD

Jason Gillihan, MD

Oscar D Guillamondegui, MD, MPH

Shannon R Kilkelly, DO

Evan Pivalizza, MBBS

e4.1) Conduct of Delphi consensus process

The Delphi was conducted as online survey. A variable was retained as confounder if more than 75% of experts expressed an agreement superior to six on a nine-point Likert Scale. All experts participated in both rounds.

DELPHI ROUND 1 -NOREPI US-F STUDY
QUESTION 1

© 2022 Gauss T et al. *JAMA Network Open.*

What are the key clinical elements leading a clinician to introduce Norepinephrine or Vasopressin within the first 6 hours of to a severe trauma patient before hemorrhage control is achieved?

Reponses provided

a) In the case of traumatic brain injury
b) In the case of spinal cord injury
c) Persistent hypotension after 1000ml of fluid expansion
d) Induction of sedation and anaesthesia
e) High Lactate > 3 mmol/l
f) Persistent hypotension after administration of 2 RBC (pre-hospital or in resuscitation room)
g) Prolonged extrication or transport time
h) Blunt cardiac injury
i) Pre-existing coronary disease
j) Pre-existing renal insufficiency

© 2022 Gauss T et al. *JAMA Network Open.*

QUESTION 2

What are predictive factors of early mortality in a severe trauma patient?

Responses provided

a) Shock as per SAP <90 mmHg

b) Shock Index > 1

c) GCS < 3

d) MGAP score < 23

e) Clinically unstable pelvic ring fracture

f) Hypoxia, SpO2 > 91%

g) Dilated pupil

h) Cardiac Arrest

i) Amputation

j) Blast mechanism

k) Traumatic coagulopathy defined by standard laboratory (INR > 1,5) or viscoelastic criteria (ROTEM: FIBTEM < 7mm/ EXTEM CA5 41mm; TEG: functional fibrinogen (FF) TEG MA 19 mm/ rTEG MA 64 mm)

l) Lactate > 4 mmol/l

m) Age > 65 years

n) pH < 7

o) Use of REBOA or thoracotomy in resuscitation room

p) Severe/uncontrollable truncal haemorrhage

q) AIS head > 3

r) ISS > 25

DELPHI ROUND 2- NOREPI-US-F STUDY

In the second round, variables identified by the experts in round 1 and available in all three datasets were submitted to expert consensus.

After Experts agreed on the following criteria for

1) Treatment allocation:

- PAS < 85 after fluid challenge expansion
- Prehospital GCS < 8 and PAS <100 mmHg

2) Confounding and adjustment variables for outcome:

Age

Sex

© 2022 Gauss T et al. *JAMA Network Open.*

Pre-admission volume expansion (will not have for US)
Pre-admission SBP
Pre-admission shock index
Pre-admission HR
Pre-admission cardiac arrest
Initial pre-hospital GCS
GCS motor score
Pre-hospital intubation
On-admission lactate
On-admission BE
On-admission Hb
On-admission SBP
On-admission shock index
On-admission HR (beats min–1)
ISS
AIS-head >3

Variables not available in the final data set were not retained for the analysis. The final results were used to generate the following Directed Acyclic Graph:

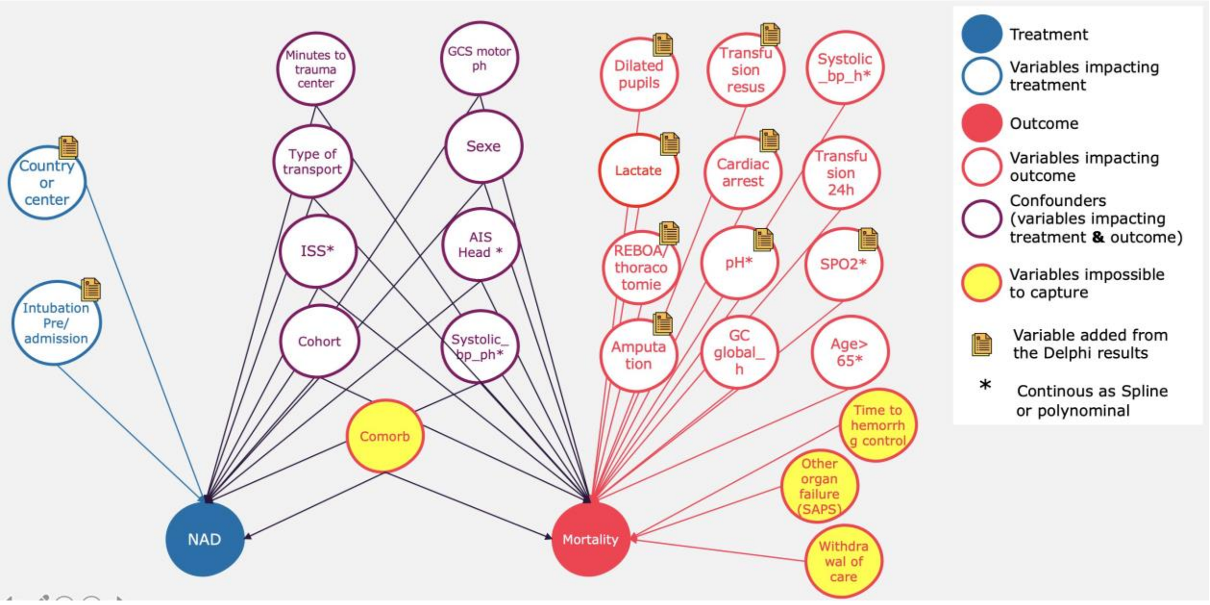

eFigure 4.1, A simplified Directed Acyclic Graph (DAG): a) pre-intervention variables associated with the decision to administer Norepinephrine (NOREPI) in purple; the same variables point towards mortality if considered a confounder. b) Explicative variables independent from treatment administration associated 24-hour mortality in red. c) Variables or confounders that the experts would have preferred to add, but were not available in yellow.
AIS= Abbreviated injury severity Score head; ISS= Injury Severity Score; GCS motor ph= prehospital Glasgow Coma Scale motor score; Systolic bp ph= prehospital systolic blood pressure; systolic bp h= systolic blood pressure in resuscitation; SpO2.min hospital=minimal peripheral oxygen saturation in resuscitation phase; pH= pH in resuscitation; Lactate= Lactate concentration in resuscitation; GCS= Glasgow Coma Scale in resuscitation

© 2022 Gauss T et al. *JAMA Network Open.*
