# Supplementary material for: Association of Early Norepinephrine Administration With 24-Hour Mortality Among Patients With Blunt Trauma and Hemorrhagic Shock
Source: JAMA Netw Open. 2022 Oct 7;5(10):e2234258. doi: 10.1001/jamanetworkopen.2022.34258 (PMC9547317; doi:10.1001/jamanetworkopen.2022.34258)
Supplement: Supplement 2. — Nonauthor Collaborators. French Trauma Research Initiative [file jamanetwopen-e2234258-s002.pdf]

*First name, last name, and suffix (if applicable) are required and will appear in PubMed.

***Group Name(s): French Trauma Research Initiative**

***First Name and Middle Initial(s)**	***Last Name**	***Suffix (eg, Jr, III)**	Academic Degrees	Institution	Location (city, state/province)	Country	Role or Contribution, eg, chair, principal investigator	Group (if more than 1 Group listed in the byline) and/or Subgroup (eg, Steering Committee)
François	Albasini		Dr	CH Saint Jean de Maurienne	Saint Jean de Maurienne	France	clinical investigator	**Trauma System of the Northern French Alps Emergency Network (TRENAU)**
Olivier	Briot		Dr	CH Briançon	Briançon	France	clinical investigator	**Trauma System of the Northern French Alps Emergency Network (TRENAU)**
Laurent	Chaboud		Dr	CH Albertville Moutiers	Albertville	France	clinical investigator	**Trauma System of the Northern French Alps Emergency Network (TRENAU)**
Sophie	Chateigner Coelsch		Dr	CH Bourg Saint Maurice	Bourg Saint Maurice	France	clinical investigator	**Trauma System of the Northern French Alps Emergency Network (TRENAU)**
Alexandre	Chaumat		Dr	CH Annecy Genevois	Annecy	France	clinical investigator	**Trauma System of the Northern French Alps Emergency Network (TRENAU)**
Thomas	Comlar		Dr	CH Métropole Savoie	Chambery	France	clinical investigator	**Trauma System of the Northern French Alps Emergency Network (TRENAU)**
Olivier	Debas		Dr	CH Docteur Recamier – Belley	Belley	France	clinical investigator	**Trauma System of the Northern French Alps Emergency Network (TRENAU)**
Guillaume	Debaty		Dr	CHU Grenoble Alpes	Grenoble	France	clinical investigator	**Trauma System of the Northern French Alps Emergency Network (TRENAU)**
Emmanuelle	Dupré-Nalet		Dr	CH Alpes Léman	Contamine sur Arve	France	clinical investigator	**Trauma System of the Northern French Alps Emergency Network (TRENAU)**
Samuel	Gay		Dr	CH Annecy Genevois	Annecy	France	clinical investigator	**Trauma System of the Northern French Alps Emergency Network (TRENAU)**
Edouard	Ginestie		Dr	CHI des Alpes du Sud – Gap	Gap	France	clinical investigator	**Trauma System of the Northern French Alps Emergency Network (TRENAU)**
Edouard	Girard		Dr	CHU Grenoble Alpes	Grenoble	France	clinical investigator	**Trauma System of the Northern French Alps Emergency Network (TRENAU)**
Jules	Grèze		Dr	CHU Grenoble Alpes	Grenoble	France	clinical investigator	**Trauma System of the Northern French Alps Emergency Network (TRENAU)**
Marc	Haesevoets		Dr	CH Albertville Moutiers	Albertville	France	clinical investigator	**Trauma System of the Northern French Alps Emergency Network (TRENAU)**
Marie	Hallain		Dr	CHI Les Hôpitaux du Pays du Mont Blanc	Sallanches	France	clinical investigator	**Trauma System of the Northern French Alps Emergency Network (TRENAU)**
Etienne	Haller		Dr	CH Saint Jean de Maurienne	Saint Jean de Maurienne	France	clinical investigator	**Trauma System of the Northern French Alps Emergency Network (TRENAU)**
Christophe	Hoareau		Dr	CH Bourg Saint Maurice	Bourg Saint Maurice	France	clinical investigator	**Trauma System of the Northern French Alps Emergency Network (TRENAU)**
Bernard	Lanaspre		Dr	CH Les Escartons – Briançon	Briançon	France	clinical investigator	**Trauma System of the Northern French Alps Emergency Network (TRENAU)**
Safia	Lespinasse		Dr	CH Pierre Bazin – Voiron	Voiron	France	clinical investigator	**Trauma System of the Northern French Alps Emergency Network (TRENAU)**
Albrice	Levrat		Dr	CH Annecy Genevois	Annecy	France	clinical investigator	**Trauma System of the Northern French Alps Emergency Network (TRENAU)**
Romain	Mermillod-Blondin		Dr	CH Annecy Genevois	Annecy	France	clinical investigator	**Trauma System of the Northern French Alps Emergency Network (TRENAU)**
Philippe	Nicoud		Dr	Les Hôpitaux du Léman – Thonon	Thonon	France	clinical investigator	**Trauma System of the Northern French Alps Emergency Network (TRENAU)**
Elisabeth	Rancurel		Dr	CHU Grenoble Alpes	Grenoble	France	clinical investigator	**Trauma System of the Northern French Alps Emergency Network (TRENAU)**
Jean-Marc	Thouret		Dr	CH Métropole Savoie	Chambery	France	clinical investigator	**Trauma System of the Northern French Alps Emergency Network (TRENAU)**
Claire	Vallenet		Dr	CH Alpes Léman	Contamine sur Arve	France	clinical investigator	**Trauma System of the Northern French Alps Emergency Network (TRENAU)**
Cécile	Vallot		Dr	CH Annecy Genevois	Annecy	France	clinical investigator	**Trauma System of the Northern French Alps Emergency Network (TRENAU)**
Bénédicte	Zerr		Dr	CH Métropole Savoie	Chambery	France	clinical investigator	**Trauma System of the Northern French Alps Emergency Network (TRENAU)**
Paér-Sélim	Abback		Dr	Tours University Hospital	Tours	France	clinical investigator	TraumaBase Group
Gérard	Audibert		Dr	Nancy University Hospital	Nancy	France	clinical investigator	TraumaBase Group
Mathieu	Boutonnet		Dr	Percy Army Hospital	Clamart	France	clinical investigator	TraumaBase Group
Thomas	Clavier		Dr	Rouen University Hospital	Rouen	France	clinical investigator	TraumaBase Group
Fabrice	Cook		Dr	Cayenne General Hospital	Cayenne	France	clinical investigator	TraumaBase Group
Mohamed	Eljamri		Dr	Hospital Group Séléstat Obernai	Séléstat	France	clinical investigator	TraumaBase Group
Thierry	Floch		Dr	Reims University Hospital	Reims	France	clinical investigator	TraumaBase Group
Elisabeth	Gaertner		Dr	Colmar Hospital	Colmar	France	clinical investigator	TraumaBase Group
Delphine	Garrigue		Dr	Lille University Hospital	Lille	France	clinical investigator	TraumaBase Group
Thomas	Geeraerts		Dr	Toulouse University Hospital	Toulouse	France	clinical investigator	TraumaBase Group
Sebastien	Gette		Dr	Regional Hospital Metz-Thionville	Metz	France	clinical investigator	TraumaBase Group
Anne	Godier		Dr	Georges Pompidou University Hospital	Paris	France	clinical investigator	TraumaBase Group
Pierre	Gosset		Dr	Amiens University Hospital	Amiens	France	clinical investigator	TraumaBase Group
Jean-Luc	Hanouz		Dr	Caen University Hospital	Caen	France	clinical investigator	TraumaBase Group

Supplemental Online Content: Nonauthor Collaborators JAMA OPEN JNO022-3829-T
*First name, last name, and suffix (if applicable) are required and will appear in PubMed.

***First Name and Middle Initial(s)**	***Last Name**	***Suffix (eg, Jr, III)**	Academic Degrees	Institution	Location (city, state/province)	Country	Role or Contribution, eg, chair, principal investigator	Group (if more than 1 Group listed in the byline) and/or Subgroup (eg, Steering Committee)					
Jean-Denis	Moyer		Dr	Beaujon University Hospital	Clichy	France	clinical investigator	TraumaBase Group					
			Dr			France	clinical investigator	TraumaBase Group					
			Dr			France	clinical investigator	TraumaBase Group					
Olivier	Langeron		Dr	Mondor University Hospital	Créteil	France	clinical investigator	TraumaBase Group					
Marc	Leone		Dr	Marseille University Hospital	Marseille	France	clinical investigator	TraumaBase Group					
Julien	Pottecher		Dr	Strasbourg University Hospital	Strasbourg	France	clinical investigator	TraumaBase Group					
Jonathan	Portaz		Dr	Wissembourg Hospital	Wissembourg	France	clinical investigator	TraumaBase Group					
Mathieu	Raux		Dr	Pitié-Salpétrière University Hospital	Strasbourg	France	clinical investigator	TraumaBase Group					
Marion	Scotto		Dr	Bordeaux University Hospital	Bordeaux	France	clinical investigator	TraumaBase Group					
Mathieu	Willig		Dr	Dijon University Hospital	Dijon	France	clinical investigator	TraumaBase Group					
Alexia	Hardy		Dr	Valenciennes Hospital	Valenciennes	France	clinical investigator	TraumaBase Group					
